# Role of BMPR2 Mutation in Lung Organoid Differentiation

**DOI:** 10.3390/biomedicines13071623

**Published:** 2025-07-02

**Authors:** Simin Jiang, Dian Chen, Liangliang Tian, Zihang Pan, Huanyu Long, Lanhe Chu, Weijing Kong, Qiyang Yao, Xiaojing Ma, Yun Zhao, Kai Wang, Yahong Chen

**Affiliations:** 1 Department of Pulmonary and Critical Care Medicine, Peking University Third Hospital, Beijing 100191, China; jsm0000@163.com (S.J.);; 2 Beijing Advanced Center of Cellular Homeostasis and Aging-Related Diseases, Clinical Stem Cell Research Center, State Key Laboratory of Vascular Homeostasis and Remodeling, Department of Physiology and Pathophysiology, School of Basic Medical Sciences, Peking University Third Hospital, Peking University, Beijing 100191, China

**Keywords:** induced pluripotent stem cell, airway organoid, alveolar organoid, pulmonary hypertension, BMPR2

## Abstract

**Background**: The bone morphogenetic protein (BMP) signaling pathway is essential for lung development. BMP4, a key regulator, binds to type I (BMPR1) and type II (BMPR2) receptors to initiate downstream signaling. While the inactivation of *Bmpr1a* and *Bmpr1b* leads to tracheoesophageal fistulae, the role of *BMPR2* mutations in lung epithelial development remains unclear. **Methods**: We generated induced pluripotent stem cells (iPSCs) from a patient carrying a BMPR2 mutation (c.631C>T), and gene-corrected isogenic controls were created using CRISPR/Cas9. These iPSCs were differentiated into lung progenitor cells and subsequently cultured to generate alveolar and airway organoids. The differentiation efficiency and epithelial lineage specification were assessed using immunofluorescence, flow cytometry, and qRT-PCR. **Results**: BMPR2-mutant iPSCs showed no impairment in forming a definitive or anterior foregut endoderm. However, a significant reduction in lung progenitor cell differentiation was observed. Further, while alveolar epithelial differentiation remained largely unaffected, airway organoids derived from BMPR2-mutant cells exhibited impaired goblet and ciliated cell development, with an increase in basal and club cell markers, indicating skewing toward undifferentiated airway cell populations. **Conclusions**: BMPR2 dysfunction selectively impairs late-stage lung progenitor specification and disrupts airway epithelial maturation, providing new insights into the developmental impacts of BMPR2 mutations.

## 1. Introduction

The development and differentiation of the lung are orchestrated by a highly conserved and intricate signaling network [1]. As a pivotal member of the transforming growth factor-beta (TGF-β) superfamily, the bone morphogenetic protein (BMP) signaling pathway plays a central role in epithelial–mesenchymal crosstalk through dynamic spatiotemporal regulation. This pathway governs key processes, including endoderm formation, lung bud branching morphogenesis, and alveolar epithelial differentiation [2,3,4,5].

During embryonic development, the lung primordium arises from the ventral foregut endoderm, with its fate determined by the intricate coordination of multiple signaling pathways. The earliest lineage restriction during embryogenesis occurs in the gastrulation stage, during which the three germ layers—endoderm, mesoderm, and ectoderm—begin to form. Activin A induces the formation of the mesoderm and endoderm in a concentration-dependent manner in both mouse and human embryonic stem cells. Low concentrations favor a mesodermal fate, whereas higher concentrations promote definitive endoderm specification [6,7]. Furthermore, the FGF and WNT signaling pathways regulate definitive endoderm differentiation based on Activin A signaling activation [8]. The cooperative action of BMP4 and Activin A also facilitates the formation of FOXA2^+^SOX17^+^ endodermal cells [9,10]. Consequently, the inhibition of BMP signaling via Noggin and the blockade of the TGF-β pathway using SB431542 induces the expression of the anterior foregut marker SOX2, while suppressing the posterior gut marker CDX2 [11]. BMP signaling gradients also play a pivotal role in the dorsal–ventral patterning of the foregut: Noggin, secreted from the dorsal mesenchyme, inhibits BMP activity to sustain high SOX2 expression, thereby promoting esophageal differentiation. Conversely, BMP4 secreted from the ventral mesenchyme activates NKX2.1 expression via BMPR1A/B receptors while repressing SOX2, initiating lung lineage specification [11]. In this context, WNT2/2b and FGF10 establish a positive feedback loop, and their functional loss results in the complete arrest of lung bud development [12,13]. Moreover, under conditions of high WNT and FGF signaling, inhibition of the TGF-β pathway combined with BMP activation has been demonstrated to effectively induce the differentiation of early lung progenitor cells into alveolar type 2 (AT2)-like cells in vitro [3].

BMP4, as a key regulatory factor in lung development, binds to type I (BMPR1) and type II receptors (BMPR2) to activate downstream signaling pathways [14]. Previous studies have shown that the inactivation of Bmpr1a and Bmpr1b leads to the loss of lung progenitor markers (Nkx2.1) and the subsequent development of tracheoesophageal fistulae in mice [15]. However, the impact of mutations in BMPR2, another core receptor of BMP4, on lung development remains unclear. In this study, we investigated the effects of BMPR2 mutations on lung development by generating airway and alveolar organoids derived from induced pluripotent stem cells (iPSCs) obtained from patients with BMPR2-related pulmonary arterial hypertension, as well as gene-corrected control iPSCs.

## 2. Materials and Methods

### 2.1. Generation of iPSC Line with BMPR2 Mutation and Gene Editing

We derived iPSCs from a female patient diagnosed with pulmonary hypertension and carrying a BMPR2 mutation (c.631C>T), provided by the Cardiovascular Department of Fuwai Hospital. A CRISPR/Cas9-corrected control iPSC line was generated as previously described in the STAR Protocol [16].

### 2.2. Differentiation of iPSCs into Lung Progenitor Cells

iPSCs were dissociated into single cells using 0.5 mM EDTA and replated at 80–90% confluence on day 0. For definitive endoderm induction (days 1–3), cells were cultured in MCDB131 medium (11.6 g/L; Thermo Fisher Scientific, Waltham, MA, USA, Cat# 10372019) supplemented with D-glucose (0.44 g/L; Sigma-Aldrich, Darmstadt, Germany, Cat# G8270), sodium bicarbonate (2.68 g/L; Thermo Fisher Scientific, Cat# S233500), BSA (5 g/L; VWR, Radnor, USA, Cat# 422351S), vitamin C (80 μg/mL; Merck, Kenilworth, NJ, USA, Cat# 100468), GlutaMAX (10 mL; Thermo Fisher Scientific, Cat# 35050061), and penicillin–streptomycin (1 mL; Sigma-Aldrich, Cat# P4333) in 1000 mL ddH_2_O. On day 1, 100 ng/mL Activin A (PeproTech, Rocky Hill, CT, USA, Cat# 120-14E) and 3 μM CHIR99021 (Selleck Chemicals, Houston, TX, USA, Cat# S2924) were added; on days 2–3, only 100 ng/mL Activin A was used. From day 4 onward, cells were cultured in DMEM/F12 (500 mL; Thermo Fisher Scientific, Cat# 11320033) supplemented with BSA (0.25 g; VWR, Cat# 422351S), vitamin C (50 μg/mL; Merck, Cat# 100468), GlutaMAX (5 mL; Thermo Fisher Scientific, Cat# 35050061), N-2 supplement (5 mL; Thermo Fisher Scientific, Cat# 17502048), B-27 supplement (10 mL; Thermo Fisher Scientific, Cat# 17504044), β-mercaptoethanol (0.04 μL/mL; Sigma-Aldrich, Cat# M3148), and penicillin–streptomycin (1 mL; Sigma-Aldrich, Cat# P4333). For anterior foregut endoderm induction (days 4–6), 2 μM dorsomorphin (Sigma-Aldrich, Cat# P5499) and 10 μM SB431542 (Tocris Bioscience, Bristol, UK, Cat# 1614) were added. For lung progenitor specification (days 7–15), cells were treated with 3 μM CHIR99021, 10 ng/mL BMP4 (PeproTech, Cat# 120-05), and 50 nM retinoic acid (Sigma-Aldrich, Cat# R2625).

### 2.3. Isolation of Lung Progenitor Cells

Lung progenitor cells can be isolated by sorting carboxypeptidase M (CPM)^+^ cells [17]. Cells were dissociated using 0.5% trypsin–EDTA (Gibco, Grand Island, NE, USA, Cat# 2053183) and filtered through a 70 μm cell strainer to obtain a single-cell suspension. To prevent nonspecific antibody binding, cells were incubated with Human TruStain FcX™ (BioLegend, San Diego, CA, USA, Cat# 422302) at 4 °C for 10 min. Subsequently, cells were stained with mouse anti-CPM antibody (R&D Systems, Minneapolis, MN, USA, Cat# MAB4616) at 4 °C for 10 min, followed by washing and incubation with PE-conjugated anti-mouse secondary antibody (BioLegend, Cat# 405307) at 4 °C for another 10 min. After washing, cells were incubated with human anti-PE nanobeads (BioLegend, Cat# 480092) at 4 °C for 15 min. PE-positive (CPM^+^) cells were isolated using a magnetic separator (BioLegend, Cat# 480019).

### 2.4. Generation of Lung Organoids

Lung progenitor cells were embedded in growth factor-reduced Matrigel (Corning, Corning, NY, USA, Cat# 354230) at a density of 400 cells/μL and cultured in DMEM/F12-based medium (as described in Section 2.2) supplemented with 3 μM CHIR99021 and 10 ng/mL KGF (PeproTech, Cat# 100-19) for one week. For alveolar organoid differentiation, cultures were treated with 3 μM CHIR99021, 10 ng/mL KGF, and DIC (50 nM dexamethasone [Sigma-Aldrich, Cat# D4902], 100 μM IBMX [Sigma-Aldrich, Cat# I5879], and 100 μM cAMP [Sigma-Aldrich, Cat# A9501]) for two weeks. Airway organoids were generated using 250 ng/mL FGF2 (PeproTech, Cat# 100-18B), 100 ng/mL FGF10 (PeproTech, Cat# 100-26), 2 μM DAPT (Sigma-Aldrich, Cat# D5942), and DIC.

### 2.5. Quantitative Real-Time PCR Analysis

Total RNA was extracted using the FastPure Cell/Tissue Total RNA Isolation Kit V2 (Vazyme, Nanjing, China, Cat# RC112). Reverse transcription was performed with the HiScript III RT SuperMix Kit (Vazyme, Cat# R323). Quantitative real-time PCR was conducted using gene-specific primers, with each sample run in technical duplicates or triplicates. Cycle threshold (CT) values were averaged, and relative gene expression was calculated using the 2^−ΔΔCT^ method, normalized to GAPDH expression. Each experiment included at least three biological replicates. The primer list is provided in Table 1.

### 2.6. Flow Cytometry Analysis

Cells or organoids were dissociated and filtered through a 40 μm cell strainer to obtain single-cell suspensions. For intracellular staining (e.g., SOX2, SOX17, NKX2.1), cells were fixed with 2% paraformaldehyde at 37 °C for 10 min and permeabilized with 0.2% Triton X-100 (Sigma-Aldrich, Cat# T8787) in PBS for 10 min. After washing and centrifugation at 300× *g* for 3 min, cells were resuspended in FACS buffer (BD Biosciences, San Jose, CA, USA, Cat# 554656) and blocked. Primary antibodies, either directly conjugated or followed by fluorescence-conjugated secondary antibodies, were used for staining at 4 °C for 20 min in the dark. For surface marker staining (e.g., CXCR4, FOXA2, MUC5AC), fixation and permeabilization were omitted. Stained cells were analyzed using a FACSAria II (BD Biosciences, Franklin Lakes, NJ, USA) or CytoFLEX flow cytometer (Beckman, Brea, CA, USA), and data were processed with the FlowJo software (v10.6.2, Ashland, OR, USA). The antibody list is provided in Table 2.

### 2.7. Immunofluorescence Staining

Cells cultured on 35 mm confocal dishes were fixed with 4% paraformaldehyde and permeabilized with 0.2% Triton X-100. After blocking with 2% BSA (VWR, Cat# 422351S) for 30 min at room temperature, cells were incubated with primary antibodies for 30 min, washed, and then incubated with fluorescence-conjugated secondary antibodies for another 30 min. Nuclei were counterstained with DAPI (Solarbio, Beijing, China, Cat# C0060, China).

### 2.8. Three-Dimensional Organoid Clearing for 3D Fluorescence Imaging

Organoids were fixed with 4% paraformaldehyde and dehydrated through a graded ethanol series (50% in PBS, 80% in ddH_2_O, 100% ethanol; 10 min each with gentle shaking). They were then washed sequentially in 20% DMSO in 80% ethanol, 80% ethanol in ddH_2_O, 50% ethanol in PBS, 100% PBS, and finally in PBS with 2% Triton X-100 for one hour. Organoids were blocked at room temperature for one hour, incubated with primary antibodies diluted in blocking buffer at 4 °C overnight, washed ten times in wash buffer, and then incubated with fluorescence-conjugated secondary antibodies for one hour. After another ten washes, nuclei were stained with DAPI (Thermo Fisher Scientific, Cat# D1306, USA). Finally, organoids were dehydrated through a graded ethanol series and embedded in CytoVista™ Tissue Clearing Reagent (Invitrogen, Carlsbad, CA, USA, Cat# V11325, USA). The details of the protocol referred to the CytoVista™ Tissue Clearing Reagent manual.

### 2.9. Statistical Analysis

Statistical analysis was performed using GraphPad Prism 9 (GraphPad software, San Diego, CA, USA). All data were expressed as the mean ± standard error of the mean (SEM). Multiple statistical comparisons within groups were performed using one-way ANOVA with Tukey’s post hoc test for multiple comparisons. Between-group comparisons were performed using unpaired Student’s *t*-tests. A *p*-value less than 0.05 was indicative of a statistically significant difference.

## 3. Results

### 3.1. Generation of Lung Progenitor Cells Using BMPR2-Mutated and Gene-Corrected iPSCs

**Figure 1:**
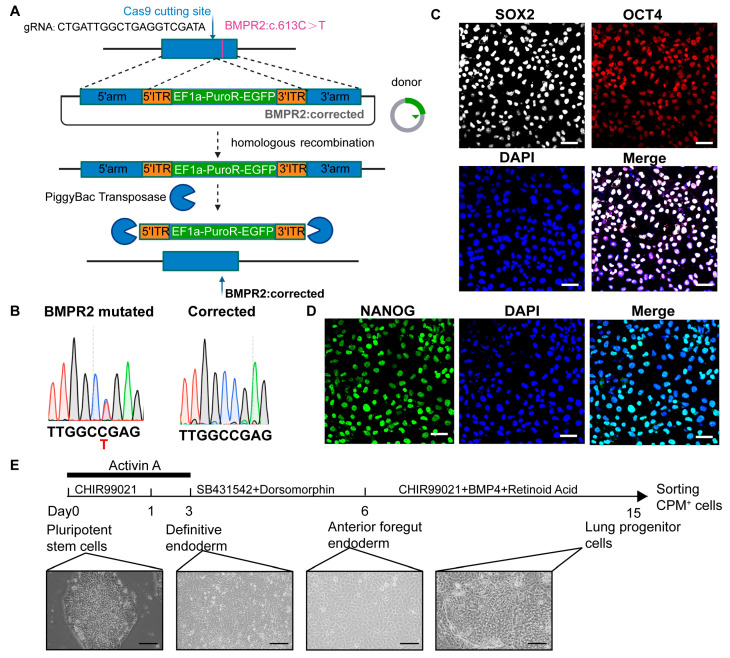
Differentiation of lung progenitor cells from induced pluripotent stem cells (iPSCs). (**A**) Schematic representation of CRISPR/Cas9-mediated correction of the BMPR2 point mutation. (**B**) iPSCs derived from a pulmonary hypertension (PH) patient carrying a BMPR2 mutation and the corresponding gene-corrected isogenic control lines. (**C**,**D**) Immunofluorescence staining confirming pluripotency marker expression in PH-iPSCs: SOX2 and OCT4 (**C**) and NANOG (**D**). Scale bar, 50 μm. *n* = 5 independent biological replicates. (**E**) Schematic of the differentiation protocol from iPSCs to lung progenitors, with representative brightfield images shown at indicated time points. Scale bar, 100 μm.

We utilized induced pluripotent stem cells (iPSCs) derived from a hereditary pulmonary hypertension (PH) patient carrying a pathogenic BMPR2 mutation (c.631C>T, p.Arg211Trp). To establish an isogenic control model, we employed CRISPR/Cas9-mediated homologous recombination (Figure 1A). Targeted sequencing analysis confirmed successful gene correction: Sanger sequencing of both the mutant iPSCs and the gene-corrected isogenic line revealed the complete restoration of the cytosine (C) to thymine (T) mutation back to the wild-type sequence in the corrected cells (Figure 1B). The pluripotency of the patient-derived iPSCs was validated by immunostaining for key pluripotency markers, including SOX2, OCT4, and NANOG (Figure 1C,D).

Building on previous protocols for the differentiation of human pluripotent stem cells (hPSCs) into lung organoids [18,19,20], we optimized the differentiation procedure (Figure 1E). Morphological transitions during differentiation were monitored via brightfield microscopy. On day 0, iPSC colonies exhibited clear boundaries and a tightly packed, rounded morphology. By days 3 and 6, the cells had transitioned into an epithelial-like monolayer with a pavement-like appearance. By day 15, lung progenitor cells had emerged, displaying an island-like morphology and forming clonal aggregates.

### 3.2. BMPR2 Mutation Reduces the Differentiation Efficiency of Lung Progenitor Cells

**Figure 2:**
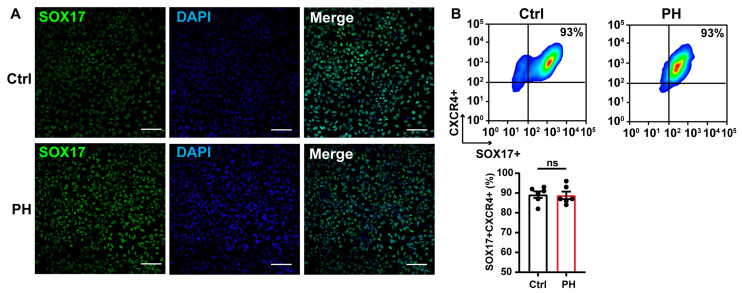
Assessment of definitive endoderm (DE) differentiation efficiency. (**A**) Immunofluorescence staining of DE marker SOX17. Scale bar, 100 μm. *n* = 3 independent biological replicates. Ctrl: control. (**B**) Representative flow cytometry image and quantification showing DE-stage differentiation efficiency (SOX17^+^, CXCR4^+^).

To investigate the impact of BMPR2 mutations on lung progenitor cell differentiation, we performed immunofluorescence staining and flow cytometry analyses at multiple stages of the differentiation process. On day 3, definitive endoderm cells were identified by SOX17 and CXCR4 co-expression (Figure 2A). Flow cytometry revealed that the differentiation efficiency in the BMPR2-mutant group (88.83 ± 1.89%) was comparable to that of the isogenic control group (89.17 ± 1.66%, *p* = 0.897) (Figure 2B), indicating that BMPR2 mutations do not affect the formation of a definitive endoderm.

**Figure 3:**
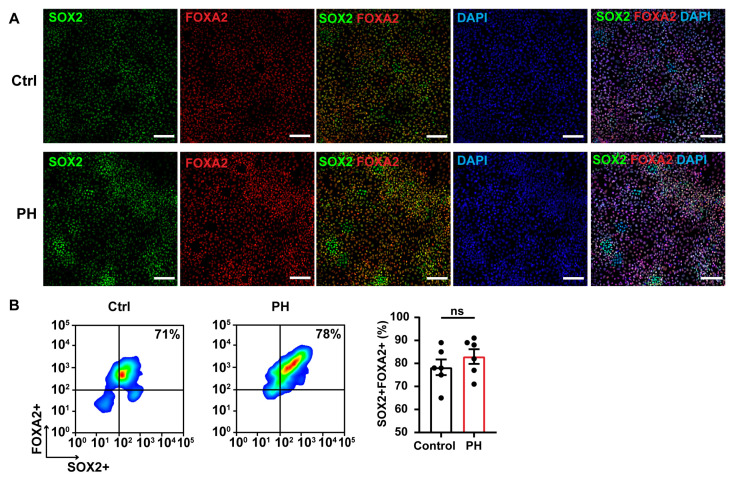
Assessment of anterior foregut endoderm (AFE) differentiation efficiency. (**A**) Immunofluorescence staining for AFE markers SOX2 and FOXA2. Scale bar, 100 μm. *n* = 3 independent biological replicates. (**B**) Representative flow cytometry image and analysis shows differentiation rates at AFE stage (FOXA2^+^, SOX2^+^).

On day 6, the anterior foregut endoderm was characterized by FOXA2 expression, while cells co-expressing FOXA2 and SOX2 were classified as primitive streak-like cells (Figure 3A). A subset of SOX2^+^FOXA2^−^ cells remained undifferentiated, representing residual pluripotent stem cells. Flow cytometry showed no significant difference in the proportion of FOXA2^+^SOX2^+^ cells between the mutant group (83.00 ± 3.19%) and the control group (78.33 ± 3.40%, *p* = 0.341) (Figure 3B).

**Figure 4:**
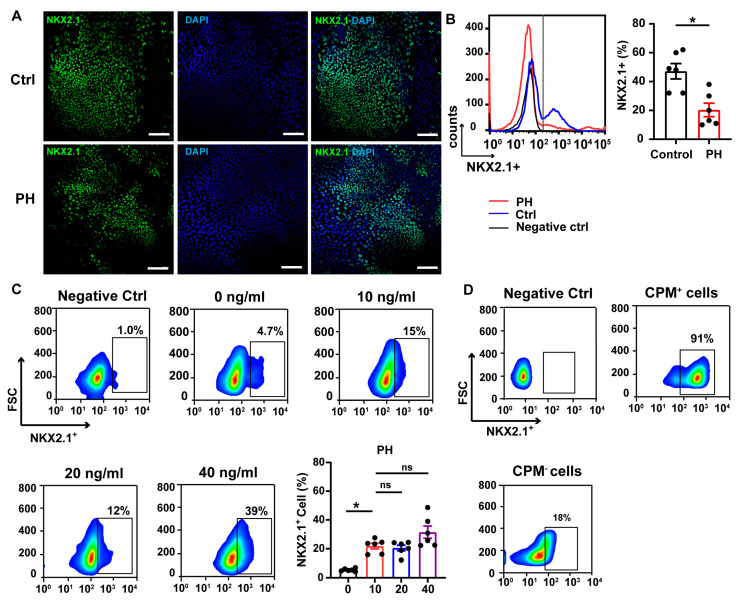
Assessment of lung progenitor cell (LP) differentiation. (**A**) Immunofluorescence staining of lung progenitor marker NKX2.1 in PH and control iPSC-derived cells. Scale bar, 100 μm. *n* = 3 independent biological replicates. (**B**) Representative flow cytometry image and analysis shows differentiation rates of LP. (**C**) Flow cytometry analysis shows lung progenitor differentiation efficiency at different BMP4 concentrations in PH group. * *p* < 0.05, unpaired Student’s *t*-test. (**D**) NKX2.1^+^ lung progenitor cells were collected by sorting CPM^+^ cells using immunomagnetic beads; sorting efficiency confirmed by flow cytometry.

By day 15, immunofluorescence staining had identified NKX2.1^+^ lung progenitor cells (Figure 4A). Flow-cytometric analysis revealed a significant reduction in differentiation efficiency in the BMPR2-mutant group (47.17 ± 5.32%) compared to the control group (70.33 ± 4.67%, *p* = 0.003) (Figure 4B). Although BMP4 depletion significantly reduced the differentiation rate of lung progenitor cells, increasing the concentration of BMP4 did not result in a statistically significant improvement in the differentiation of anterior foregut cells into lung progenitor cells in the PH group. However, treatment with 40 ng/mL BMP4 showed a trend toward increased differentiation efficiency, though this increase was not statistically significant (Figure 1C). These findings suggest that BMP4 is crucial for the differentiation of anterior foregut cells into lung progenitors, and that BMPR2 loss-of-function disrupts this process, thereby reducing the overall differentiation efficiency.

To further enrich the lung progenitor cells, we employed magnetic-activated cell sorting (MACS) using CPM as a surface marker (Figure 4D). Three cell populations were analyzed: unsorted cells, CPM^+^ sorted cells, and the CPM^−^ discarded fraction. Flow cytometry showed that the CPM^+^ population was highly enriched for NKX2.1^+^ cells (~91%), while the discarded fraction contained only ~18% NKX2.1^+^ cells, confirming the effectiveness of CPM-based purification.

**Figure 5:**
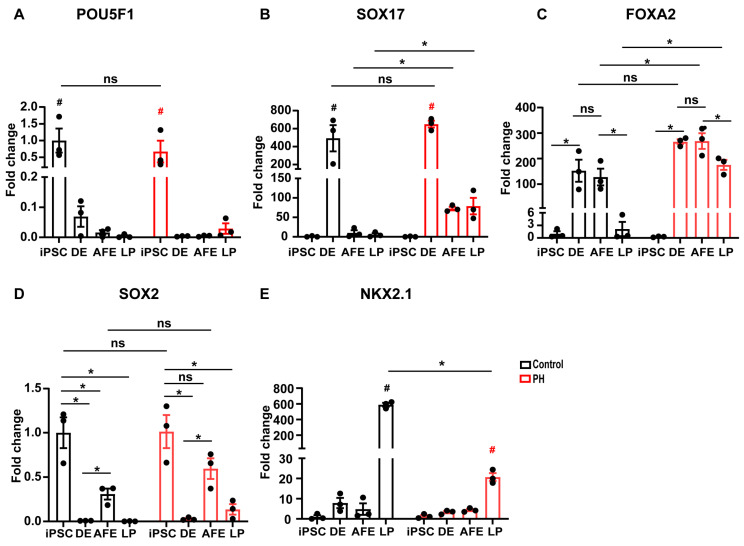
qRT-PCR analysis of key marker expression across differentiation stages. Relative mRNA expression levels of markers POU5F1 (**A**), SOX17 (**B**), FOXA2 (**C**), SOX2 (**D**), and NKX2.1 (**E**) in control and PH groups across the hPSC, DE, AFE, and LP stages. Data are presented as mean ± SE. * *p* < 0.05, unpaired Student’s *t*-test. ^#^ *p* < 0.05, two-way ANOVA with Tukey’s post hoc analysis.

We also performed quantitative real-time PCR (qRT-PCR) to assess the expression dynamics of key lineage markers throughout the differentiation process (Figure 5). As expected, the expression of POU5F1 (OCT4) was the highest at the pluripotent stage and decreased progressively during differentiation (Figure 5A). SOX17 was minimally expressed in pluripotent cells but peaked during the definitive endoderm stage and declined thereafter (Figure 5B). FOXA2 expression increased significantly during the definitive endoderm and anterior foregut stages (Figure 5C). SOX2 showed a biphasic pattern: high expression in pluripotent stem cells, reduced expression during definitive endoderm formation, and re-elevation during the anterior foregut stage due to its role in primitive streak cell identity, followed by a decline as cells committed to the lung fate (Figure 5D). NKX2.1, a lung progenitor-specific marker, exhibited marked upregulation on day 15, confirming the successful lung lineage specification (Figure 5E).

Importantly, BMPR2 mutations did not affect iPSC pluripotency, as no significant differences were observed in OCT4 and SOX2 expression between the PH-derived and control iPSC lines. Similarly, no significant differences were found in SOX17 expression at the definitive endoderm stage or SOX2 expression at the anterior foregut stage. However, NKX2.1 expression was significantly reduced in the PH group, indicating that BMPR2 mutations specifically impair the final transition to lung progenitor cells. Collectively, these results demonstrate that BMPR2 dysfunction selectively disrupts the late-stage differentiation of iPSCs into lung progenitors.

### 3.3. BMPR2 Mutation Affected the Differentiation of Airway Organoids

**Figure 6:**
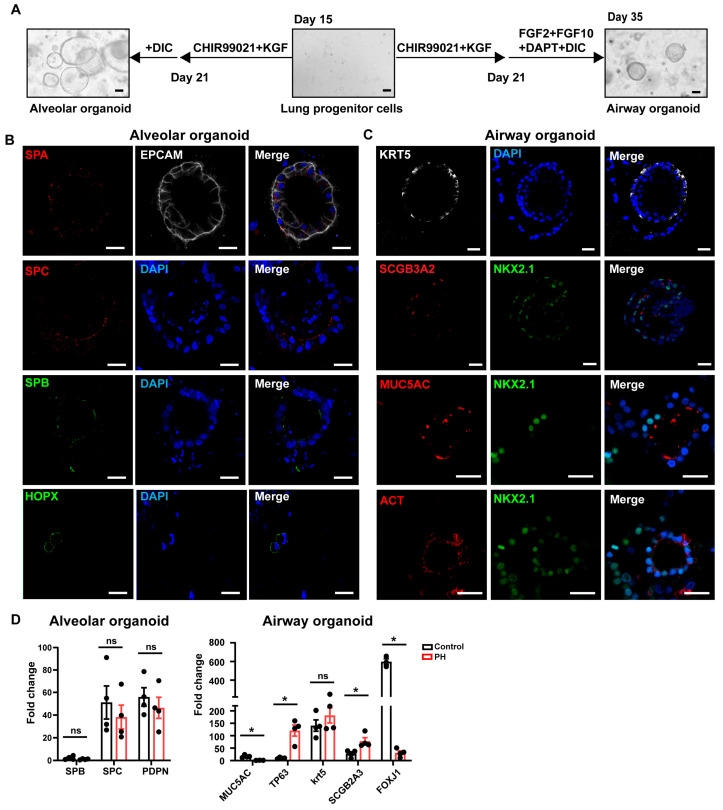
Impacts of BMPR2 mutation on airway lineage differentiation. (**A**) Schematic of the differentiation protocol from lung progenitors to alveolar and airway organoids, with representative brightfield images shown at indicated time points. Scale bar, 100 μm. (**B**) Characterization of alveolar organoids in PH group: alveolar type I cells (HOPX^+^), alveolar type II cells (SPA^+^, SPB^+^, SPC^+^), and lung epithelial cells (EPCAM^+^). Scale bar, 25 μm. (**C**) Characterization of airway organoids in PH group, including basal cells (KRT5^+^), club cells (SCGB3A2^+^), and goblet cells (MUC5AC^+^). Scale bar, 25 μm. (**D**) qRT-PCR analysis of marker expression in alveolar and airway organoids from PH and control groups. * *p* < 0.05, unpaired Student’s *t*-test.

We examined how the BMPR2 mutation affected the differentiation of lung progenitor cells into alveolar and airway organoids. Purified lung progenitor cells were embedded in growth factor-reduced Matrigel to create a 3D environment for directed differentiation. For the initial 7-day expansion, cells were cultured with 3 μM CHIR99021 and 10 ng/mL KGF (Figure 6A).

This was followed by treatment with a differentiation cocktail (100 nM dexamethasone, 0.1 mM IBMX, 10 μM cAMP) for 14 days to induce alveolar epithelial maturation. By day 35, immunofluorescence staining confirmed the presence of SPA^+^, SPB^+^, and/or SPC^+^ alveolar type II (AT2) cells and HOPX^+^ alveolar type I (AT1) cells (Figure 6B). We also stained EPCAM, which is expressed in epithelial cells. A distinct protocol was used to generate airway organoids. After 7 days of expansion, CHIR99021 and KGF were removed, and cells were cultured with FGF2, FGF10, DAPT, and the DIC cocktail for 14 more days. Immunofluorescence staining revealed SCGB3A2^+^ club cells, KRT5^+^ basal cells, MUC5AC^+^ goblet cells, and ACT^+^ ciliated cells (Figure 6C). The 3D reconstruction of the airway organoid with goblet cells is shown in Video S1. Immunofluorescence staining of airway and alveolar organoids confirmed that the BMPR2-mutated iPSCs retained the ability to differentiate into key pulmonary epithelial cell types in vitro.

The qPCR on day 35 showed that the BMPR2 mutation did not affect AT1 or AT2 cell formation. However, it significantly impaired goblet and ciliated cell differentiation, while increasing basal and club cell marker expression. These findings suggest that BMPR2 mutations disrupt airway epithelial maturation by skewing differentiation toward stem-like cell populations (Figure 6D).

## 4. Discussion

In this study, we investigated the impact of BMPR2 mutations on lung development by generating and analyzing airway and alveolar organoids derived from iPSCs obtained from a patient with BMPR2-related pulmonary hypertension (PH) and gene-corrected control iPSCs. Our results demonstrated that BMPR2 mutations impair the differentiation efficiency of lung progenitor cells, particularly affecting the transition from anterior foregut cells to lung progenitor cells. Moreover, we found that, while BMPR2 mutations did not significantly alter the generation of AT1 and AT2 cells in alveolar organoids, they notably decreased the differentiation of goblet and ciliated cells while increasing the markers for basal and club cells in airway organoids.

PH is a progressive disease characterized by a resting mean pulmonary arterial pressure (PAP) of ≥20 mm Hg [21]. Its key pathological features include excessive vascular remodeling, endothelial dysfunction, dysregulated apoptosis, and the proliferation of pulmonary arterial smooth muscle cells [22]. BMPR2 is the most commonly mutated gene in PH [23]; consequently, prior studies have primarily focused on its role in the pulmonary vasculature [24,25,26]. However, the impact of BMPR2 loss on lung epithelial development remains poorly understood. Multiple studies have shown that reduced BMPR2 signaling promotes endothelial–mesenchymal transition (EndMT) in pulmonary arterial endothelial cells [27,28,29,30]. At the same time, BMPR2 downregulation facilitates epithelial–mesenchymal transition (EMT) by amplifying TGF-β–SMAD2/3 signaling, which drives EMT more aggressively [31]. Additionally, BMPR2 loss disrupts the normal crosstalk between the BMP and Wnt signaling pathways that is essential in maintaining epithelial identity. Specifically, the ability of BMP to repress Wnt/β-catenin-driven mesenchymal gene expression is weakened [32]. The reduced differentiation efficiency of lung progenitor cells observed in the PH group may therefore result from increased mesenchymal transition and the impaired onset of EMT.

Lung epithelial cells can undergo epithelial–mesenchymal transition (EMT) and give rise to myofibroblasts, contributing to fibrotic remodeling [33]. The BMP antagonists Gremlin and Follistatin-like 1 (Fstl1) are consistently upregulated in fibrotic diseases, where they inhibit BMP signaling and unleash EMT, promoting myofibroblast accumulation and fibrosis [34,35,36,37]. The therapeutic restoration of BMPR2 signaling, or the pharmacological inhibition of downstream EMT drivers, may help to rebalance epithelial and mesenchymal programs. Interestingly, BMP signaling can also facilitate EMT in normal airway epithelial cells during epithelial restitution, a process that supports wound repair [38]. In our study, BMPR2 mutations selectively impaired airway epithelial differentiation, resulting in a significant reduction in goblet and ciliated cell populations, alongside the expansion of basal and club cell markers. This shift in cell type composition suggests that the loss of BMPR2 stalls airway epithelial cells in an undifferentiated, progenitor-like state. Since goblet and ciliated cells are essential for effective mucociliary clearance, their depletion likely compromises airway defense mechanisms—a hallmark of many chronic pulmonary disorders [39]. Conversely, the expansion of basal and club cells may reflect either a compensatory response to impaired differentiation or an early stage of airway remodeling and fibrogenesis. Notably, alveolar organoid differentiation remained unaffected, which can be attributed to the continuous activation of Wnt signaling by CHIR99021—a selective glycogen synthase kinase-3 (GSK-3) inhibitor—during alveolar culture. This treatment is known to promote the proliferation and maturation of alveolar progenitors [40,41]. In contrast, CHIR99021 is withdrawn during airway organoid differentiation, thereby unmasking the critical requirement for BMPR2 signaling in airway lineage maturation. This protocol-dependent difference likely explains why BMPR2 downregulation selectively disrupts airway, but not alveolar, differentiation.

Our study provides new insights into the role of BMPR2 mutations in lung epithelial development and a potential epithelial contribution to the pathogenesis of PH. Although asymptomatic carriers of BMPR2 mutations have a markedly elevated risk of developing PH, disease penetrance remains incomplete, with only 20–30% of carriers eventually manifesting the condition [42,43,44,45]. This incomplete penetrance is likely influenced by a combination of genetic, epigenetic, and environmental modifiers [42]. Given the close crosstalk between the lung epithelium and vasculature, which plays a crucial role in PH pathogenesis [46], epithelial defects arising from BMPR2 dysfunction, such as increased susceptibility to epithelial–mesenchymal transition (EMT), may predispose certain individuals to disease development under permissive conditions. This mechanism may help to account for the variable clinical expression observed among BMPR2 mutation carriers.

There are several limitations to this study. First, the iPSC-derived organoid model is based on a single patient line with an isogenic gene-corrected control, which may limit the generalizability of the findings due to the absence of inter-individual variability. Second, the iPSC-derived lung organoids were generated using a simplified in vitro approach, whereas lung development in vivo is orchestrated by far more complex and dynamic factors. While this model recapitulates key aspects of lung development and differentiation, its relatively homogeneous cellular composition and lack of a physiologically relevant microenvironment—including immune components, a vascular network, and an organized extracellular matrix—may constrain the external validity of the results. Third, epithelial differentiation was primarily assessed using qPCR. Although immunofluorescence analysis was performed, quantification across organoids was limited due to the inherent heterogeneity in lineage differentiation within individual organoids. A more extensive dataset would be necessary for the robust statistical evaluation of epithelial cell populations.

## 5. Conclusions

In conclusion, our study provides novel insights into the role of BMPR2 in lung organoid differentiation, highlighting its critical function in airway maturation. These findings deepen our understanding of the molecular mechanisms underlying BMPR2-related lung pathologies and may inform therapeutic strategies targeting BMP signaling to mitigate disease progression.

## Figures and Tables

**Table 1:** Primer list.

Primer List	Forward Sequence (5′ to 3′)	Reverse Sequence (5′ to 3′)
GAPDH	GGTGTGAACCATGAGAAGTATGA	GAGTCCTTCCACGATACCAAAG
NKX2.1	CGGCATGAACATGAGCGGCAT	GCCGACAGGTACTTCTGTTGCTTG
SOX17	GTGGACCGCACGGAATTTG	GGAGATTCACACCGGAGTCA
SOX2	TGGACAGTTACGCGCACAT	CGAGTAGGACATGCTGTAGGT
FOXA2	GGAACACCACTACGCCTTCAAC	AGTGCATCACCTGTTCGTAGGC
POU5F1	GGGCTCTCCCATGCATTCAAAC	CACCTTCCCTCCAACCAGTTGC
SPC	CACCTGAAACGCCTTCTTATCG	TTTCTGGCTCATGTGGAGACC
SPB	TGTCCTCCGATGTTCCACTGAG	AGCCTGTTCACTGGTGTTCCAG
MUC5AC	CATCTGCCAGCTGATTCTGA	AAGACGCAGCCCTCATAGAA
FOXJ1	CCTGTCGGCCATCTACAAGT	AGACAGGTTGTGGCGGATT
PDPN	TCCAGGAACCAGCGAAGAC	CGTGGACTGTGCTTTCTGA
TP63	ACTGCCAAATTGCAAAGACA	TGACTAGGAGGGGCAATCTG
KRT5	GAGCTGAGAAACATGCAGGA	TCTCAGCAGTGGTACGCTTG
SCGB3A2	GGCTAAGGAAGTGTGTAAATGAGC	CCATCCACCTCCGCTCTTTATC

**Table 2:** Antibody list.

Primary Antibody	Company	Location	CAT
Mouse anti-OCT4	Cell Signaling Technology	Danvers, MA, USA	75463
Rabbit anti-NANOG	Cell Signaling Technology	Danvers, MA, USA	4903
Rabbit anti-SOX2	Santa Cruz Biotechnology	Dallas, TX, USA	sc-8344
Mouse anti-SPC	Santa Cruz Biotechnology	Dallas, TX, USA	sc-518029
Rabbit anti-SPB	Abcam	Cambridge, UK	Ab271345
Rabbit anti-SPA1	Abclonal	Wuhan, China	A3133
Mouse anti-SOX2	Thermo Fisher Scientific	Waltham, MA, USA	MA1-014
Rabbit anti-HOPX	Abclonal	Wuhan, China	A15537PM
Rabbit anti-FOXA2	Abcam	Cambridge, UK	ab108422
Mouse anti-MUC5AC	Thermo Fisher Scientific	Waltham, MA, USA	MA5-12178
Rabbit anti-SCGB3A2	Abcam	Cambridge, UK	ab181853
Rabbit anti-KRT5	Cell Signaling Technology	Danvers, MA, USA	71536S
Rabbit anti-NKX2.1	Abcam	Cambridge, UK	ab76013
Mouse anti-ACT	Santa Cruz Biotechnology	Dallas, TX, USA	sc-8432
Mouse anti-CPM	Novus Biologicals	Littleton, CO, USA	DDX0520P
PE anti-SOX17	BD Biosciences	Franklin Lakes, NJ, USA	561591
APC anti-CXCR4	BD Biosciences	Franklin Lakes, NJ, USA	555976
**Secondary Antibody**	**Company**	**Location**	**CAT**
Donkey anti-rabbit IgG, Alexa Fluor 568	Thermo Fisher Scientific	Waltham, MA, USA	A10042
Donkey anti-rabbit IgG, Alexa Fluor 647	Thermo Fisher Scientific	Waltham, MA, USA	A-31573
Donkey anti-goat IgG, Alexa Fluor 488	Thermo Fisher Scientific	Waltham, MA, USA	A-11055
Donkey anti-goat IgG, Alexa Fluor 594	Thermo Fisher Scientific	Waltham, MA, USA	A-11058
Donkey anti- goat IgG, Alexa Fluor 647	Thermo Fisher Scientific	Waltham, MA, USA	A-21447

## Data Availability

The original contributions presented in this study are included in the article. Further inquiries can be directed to the corresponding authors.

## Supplementary Materials

The following supporting information can be downloaded at: https://www.mdpi.com/article/10.3390/biomedicines13071623/s1, Video S1: The 3D architecture of airway organoids includes Goblet cells. The image shows MUC5AC (red), NKX2.1 (green), and DAPI (blue) staining.

## Abbreviations

The following abbreviations are used in this manuscript:

PH	Pulmonary hypertension
AT1	Alveolar type I cells
AT2	Alveolar type I cells
AFE	Anterior foregut endoderm
Ctrl	Control
DE	Definitive endoderm
EMT	Epithelial–mesenchymal transition
EndMT	Endothelial–mesenchymal transition
LP	Lung progenitor
iPSC	Induced pluripotent stem cell
